# A mini review of what matters in the management of NAS, is ESC the best care?

**DOI:** 10.3389/fped.2023.1239107

**Published:** 2023-07-14

**Authors:** Enrique Gomez Pomar

**Affiliations:** ^1^ Division of Neonatology, Department of Pediatrics, University of Kentucky, Lexington, KY, United States; ^2^ Department of Pediatrics, St. Bernards Regional Medical Center, Jonesboro, AR, United States

**Keywords:** NAS, ESC, opioid withdrawal, neonatal withdrawal, rooming-in

## Abstract

As the use of opioids and polysubstance by pregnant women has increased over the years, there has also been a sharp increase in cases of neonatal abstinence syndrome (NAS). Classically, infants affected by NAS have been cared for in neonatal intensive care units resulting in an increase of healthcare expenditure and resource utilization as well as separation from the families. Consequently, the Eat, Sleep, and Console (ESC) tool was developed and promoted as a novel method that focuses on maternal/infant dyad during hospital stay while decreasing the use of pharmacological interventions and therefore decreasing the length of stay and healthcare expenditure. Thus, it has been implemented in several hospitals in the United States. Although the training of staff has been proposed and the interventions of sleep, eat, and console are defined, there still exists a lack of standardization of this practice specifically in regard to the type of associated non-pharmacological practices as well as the reports of its short- and long-term outcomes.

## Introduction

Neonatal abstinence syndrome (NAS) refers to a constellation of signs experienced by some newborns born from mothers that have used opioids during pregnancy. Because of the opioid epidemic and the increase in the number of infants born following *in-utero* opioid exposure, NAS has recently been referred to as neonatal opioid withdrawal syndrome (NOWS). For the purpose of this perspective on Eat, Sleep, and Console (ESC), the term NAS would be more appropriate since most of those with opioid use disorders also use other drugs, and withdrawal symptomatology may not all be attributable to opioid withdrawal. NAS occurs when there is an abrupt termination of the trans-placental transfer of addictive substances. Maternal opioid use and its effects on newborns were first described in 1875, and its incidence has dramatically increased over the years (1). From 2010 to 2017, maternal opioid use increased from 3.5 to 8.2 per 1,000 delivery hospitalizations resulting in an increase in NAS cases from 4 to 7.3 per 1,000 birth hospitalizations (2).

## Assessment and management of infants with NAS

The evaluation and management of NAS also remains non-standardized. Studies showed and experts recommend that each institution develop a protocol establishing the prenatal screening for drug use, the postnatal follow-up of infants at risk, and, for those affected, the use of non-pharmacological measures while promoting rooming-in as much as possible (3, 4). Tools to objectively evaluate infants at risk for NAS have been developed over the years, and the Finnegan NAS (FNAS) tool remains the most used (3). Once affected infants are identified to need pharmacological treatment; despite the use of non-pharmacological measures, it is recommended that these infants are monitored for cardiorespiratory events (1, 5, 6).

Infants with NAS are often admitted to the NICU for pharmacological treatment; however, this model resulted in increased costs and length of stay (LOS) (7). Therefore, a novel model called the eat, sleep, and console (ESC) was proposed wherein infants stayed with their parents during the entire hospitalization, and pharmacological intervention was given only if the infant was inconsolable, not eating or sleeping adequately (8). This initiative only focuses on three outcomes: decrease pharmacological intervention, decrease LOS, and decrease hospital cost, while lacking in standardized approaches regarding pharmacological and non-pharmacological interventions or as basic as to what constitutes adequate feeding in infants with NAS (1, 8–10). Since then, multiple institutions have adopted the ESC model without considering any clinical outcomes or short- and long-term consequences on these infants (11–15).

The ESC tool focuses, as its name suggests, on the ability of the neonate to eat, sleep, and be consoled. If the infant was able to breastfeed effectively or take ≥ 1 oz from a bottle per feed, to sleep undisturbed for ≥ 1 h, and, if crying, to be consoled within 10 min, then the infant was deemed to be well. Otherwise, non-pharmacological interventions were maximized and, if unsuccessful, then morphine was initiated or increased (8). Subsequent evaluation of the implementation of the ESC tool shows that most reports are from quality improvement (QI) projects focusing on LOS, rate of medication use, non-pharmacological care, and limited use of the FNAS (15).

There are important variations in the way that the ESC tool is implemented, even within the QI reports (16). Variations are found on the “assessment method” [the original study used ≥ 1 oz of feeding volume as successful (8), which is different from others (14, 17, 18)] and in areas as important as proposed medications and dosages. To consider, the ranges of the initial dose of morphine went from 0.03 to 0.1 mg/kg/dose (6, 14, 19), and the original ESC report used 0.05 mg/kg/dose (8) (Table 1).

**Table 1:** Comparison of studies on the “Eat, Sleep, and Console” approach in the management and assessment of infants with NAS.

	Grossman et al. (8)	Blount et al. (19)	Dodds et al. (14)	Miller and Willier (11)	Ryan et al. (6)	Amin et al. (17)	Young et al. (18)
Number of patients	287	76	82	135	158	71	837
Setting	Urban	Not specified	Urban	Urban	Rural/urban	Rural	Urban
MOUD	100%	∼85%	Not specified	∼94%	Not specified	Not specified	∼73%
Eat	1 oz	>1 oz	Breastfed well or took the prescribed amount of formula	0.5–1 oz	Breastfeed well or took >1 oz	0.5–1 oz	Ability to coordinate feeding (breast or bottle) within 10 min, breastfeeding ≥ 10 min and taking ≥ 10 ml
Sleep	>1 h	>1 h	>1 h	>1 h	>1 h	>1 h	>1 h
Console	Within 10 min	Within 10 min	Within 10 min	Within 10 min	Within 10 min	Within 10 min	Within 10 min
Pharmacological intervention	Morphine 0.05 mg/kg/dose	Morphine 0.03 mg/kg/dose	Morphine 0.1 mg/kg/dose	Methadone	Morphine 0.04 mg/kg/dose	Methadone	Per institution
Short-term outcomes	30-day readmissions	Percent weight change 30-day readmission	Readmission rate	30-day readmission	30-day readmission	30-day readmission	3-months composite safety measure

Recently, a cluster randomized clinical trial was published comparing ESC vs. the traditional management of infants with NAS (18). Initially, the trial involved 26 US sites and enrolled 1,305 infants. Only 837 infants met *a priori* definition of primary outcome of birth to discharge duration, and other outcomes included LOS, pharmacological interventions, and monitoring of adverse outcomes up to 3 months of life from various sources. The study showed a shorter readiness for discharge [8.2 vs. 14.9 days; adjusted mean difference, 6.7 days; 95% confidence interval (CI), 4.7–8.8] when the ESC model was implemented. The proportion of infants who received opioid treatment was 52.0% in the usual-care group and 19.5% in the ESC group (absolute difference, 32.5 percentage points; relative risk, 0.38; 95% CI, 0.30–0.47). Of notice, the trial showed that ∼60% of the participants were exposed to polysubstance; however, most of the participants were in a medications for opioid disorder (MOUD)” program (∼70%). In addition, 83% and 91% of the participants, respectively, were in the metropolitan area in the pre- and post-intervention groups (18).

Even though this trial is the first to prospectively and objectively analyze the management of NAS under the ESC model and showed consistent results regarding LOS and use of pharmacological agents, it does mention heterogeneity of treatment effect due to multiple factors: location of care and feeding regimen. The trial defined “Eat” as the ability to coordinate feed (breast or bottle) within 10 min, breastfeeding ≥ 10 min and taking ≥ 10 ml. The non-pharmacological interventions were also dependent on the available resources of each site. Moreover, there were variations among individual sites as to the choice of pharmacotherapy (type, dose, and use of adjunct medications) (18).

## Short- and long-term effects of NAS

Infants born of mothers who used opioids were reported to be at increased risk for prematurity, small for gestational age, NICU admission lower 5-min APGAR scores (20), and smaller head circumference and body length. Infants with *in-utero* opioid exposure have higher mortality (21, 22) compared with non-exposed infants. A retrospective analysis of 864 infants with NAS showed that infants had growth retardation between birth and discharge in all parameters with no improvement despite increased caloric content (23). Affected infants also have decreased feeding efficiency, more apneic swallows, and less respiratory rhythmic stability (24). Dysregulations in the growth curve have been tracked into adulthood (25).

Studies looking at the neurodevelopmental outcomes in newborns during the first days of life show conflicting data (26). Early signs of NAS include motor and autonomic dysregulations, manifested as lower quality of movements, lower self-regulation, and higher levels of arousal; however, the reports are inconsistent as whether this dysregulation results in neurodevelopmental impairments after the first week of life (26). On the other hand, a study published in 2020 showed that infants being treated pharmacologically for NAS had more adverse cardiorespiratory events compared with those who were not treated (5), which correlated with the reported changes in heart rate variability and autonomic stability (27). Also, it has been established that infants affected by NAS are at an increased risk of hospital readmissions (most commonly due to respiratory issues) and to require intensive care treatment (28) with increased risk of mortality (29).

Long-term negative effects in the outcome of infants with NAS were initially described as early as 1973, noticing behavioral disturbances and growth impairment (30). A systematic review that examined 79 studies including infants with polysubstance exposure showed that infants and toddlers performed worse on tests of cognitive and motor skills and on behavioral assessments (31). This finding has been reported in other studies as well; however, neurodevelopmental outcomes are also influenced by many other factors that must be taken into consideration (26, 32, 33).

As the child grows, there have been reports of differences in IQ and in the neurological and language performance; however, most of these differences disappear when other confounders are controlled (26, 31). Even though the cognitive outcomes have inconsistent findings in the studies reporting long-term effects of NAS, there are also similar inconsistencies in related behavioral outcomes, and therefore follow-up of exposed children is crucial (26).

Short- or long- term consequences of the ESC tool specifically have not been studied. Some reports indicate that weight loss increased with its implementation (11, 19), and another report stated that no differences in weight loss after implementation; however, discharge weight was obtained only at day 5 of life (11). Some of the reports include re-hospitalization rate at 30 days post-discharge; however, this rate was only reported for NAS-related admissions (8, 12, 14, 19). Young et al. reported outcomes after hospital discharge that were assessed at 3 months of age by means of a review of electronic medical records and media review through a search of public records. An evaluation of the neurodevelopmental outcomes at 2 years of age is included in the protocol (18).

A retrospective review comparing FNAS and ESC tool focused on the correlation between both methods during the implementation of the ESC model in three hospital settings (6). A receiver operating characteristic analysis showed that an FNAS cutoff of 7.5 corresponded to at least one negative component on the ESC tool (sensitivity = 0.84, specificity = 0.70, area under the curve = 0.842), which indicates the need for pharmacological intervention. This correlation has been reported by others (17). The study also found an increase in the care of infant/mother dyads in the community hospital rather than at the referral center as well as more maternal referrals for substance abuse treatment after the implementation of the method, consistent with a more family-oriented intervention (6). However, lack of proper communication can be an issue as shown in a study by McRae et al. looking at parental perspectives of the ESC model in which inadequate communication and support of the parents created feelings of guilt, fear, and stress as well as uncertainty in what happens after delivery (34).

Since the ESC model has gained much momentum despite its lack of well-controlled studies, standardized assessment, and management, careful consideration must be made as to other factors that may have an impact on the LOS and initiation of pharmacological treatment. Advocates of the ESC model refer to parental involvement in NAS care as something novel (8, 35); however, long before the ESC tool was proposed, the study by Holmes et al. focusing on rooming-in and parental involvement reported similar outcomes (36), i.e., shorter length of stay and decrease the need for pharmacological treatment. This QI project was conducted at a rural academic tertiary center focusing on standardization of the assessment and management of these infants by training of the staff (nurses and providers) on how NAS symptoms affected individual infants as well as implementing rooming-in, family support, and education. The authors reported a decrease in the cumulative dose of morphine (from 13.7 to 6.6 mg) as well as LOS (from 16.9 to 12.3 days) and therefore, an associated decrease in hospitalization costs. The authors did not report increased readmission rate during the 30 days after discharge and further, follow-up of newborns with high scores, and no treatment did not show complications at 1–4 months after discharge (36).

On the other hand, none of the ESC QI reports have evaluated what non-pharmacological interventions are most effective and how to standardize them. Even as early as 1974, non-pharmacological interventions have been described and recommended as an approach to the management of withdrawal manifestations noted from a comprehensive assessment of infants with NAS (37). A 2018 systematic review focusing on rooming-in found consistent evidence that rooming-in reduces both the use of pharmacological intervention as well as LOS (38). However, a retrospective review from the Appalachian region after the implementation of the ESC model showed no significant changes in the number of infants needing pharmacotherapy or the length of stay (17). This study found that in 41% of NAS cases, there was not a parent present, and their presence decreased further in infants that required pharmacotherapy, which could explain their results (17). Others have reported that a lack of parental involvement was significantly correlated to the need for pharmacological intervention (11). Considering these results, we can infer that the key to a successful model that manages infants affected by NAS must consider the environmental settings where the mother/infant dyad resides. A large proportion of mothers with opioid use disorder from rural areas of the country encounter inherent barriers regarding the availability and access to healthcare (1). Considering that rooming-in seems to be the common determinant to a successful management of these babies (6, 8, 10, 14, 15, 36, 38) with NAS, the question arises as to what happens in the areas in where access to healthcare and rooming-in is more difficult.

Infants affected by NAS are at risk of multiple factors, including the effects of maternal co-morbidities, types of drugs used, and socio-cultural determinants, as well as a myriad of changes in the autonomic stability and the neurodevelopmental outcomes of the infant (16, 17, 39). Public health efforts aiming to increase access to antenatal counseling and treatment are needed as well as standardization of the care for the neonate affected by maternal drug use (1, 16). To date, there is no consisted approach to the ESC tool, and success to its implementation should not only be measured as to the reduction in the LOS and hospital cost. However, rooming-in paired with intensive non-pharmacological interventions seems to have consistent positive results in the management of infants affected by NAS (38). There are a myriad of signs in NAS, besides disorganized sleep, poor disorganized or dysfunctional feeding, and irritability. Additional or other ways of non-pharmacological approach would be needed to minimize the other signs not addressed by the ESC.

## Conclusion

The interventions that appear to ameliorate the effects of maternal drug use include the following: access to prenatal care as well as to treatment programs and mitigation of polysubstance use (40, 41) paired with identification and intervention of adverse Social Determinants of Health (1, 17, 32); for hospitals that manage infants with NAS, an established protocol (1, 4) to define, promptly initiate and reinforce non-pharmacological interventions, and, if needed, continuous cardiorespiratory monitoring of infants that require pharmacotherapy (1, 19) while facilitating rooming-in (38) and breastfeeding when appropriate (42); and prompt follow-up needs to be established to ensure that any nutritional, growth, and/or neurodevelopmental challenges are rapidly identified and intervened (32, 42). Further research needs to standardize the outcome data to provide more homogenous results to compare different interventions for the management of maternal drug use and infants affected by NAS (43). Currently, standardization of ESC method is needed. Furthermore, there have been no research studies that have adequately evaluated the short- and long- term outcomes of the ESC tool (1, 16).
